# My hands are running away – learning a complex nursing skill via virtual reality simulation: a randomised mixed methods study

**DOI:** 10.1186/s12912-023-01384-9

**Published:** 2023-06-27

**Authors:** Christian Plotzky, Barbara Loessl, Barbara Kuhnert, Nina Friedrich, Christiane Kugler, Peter König, Christophe Kunze

**Affiliations:** 1 Faculty Health, Safety, Society, Care & Technology Lab, Furtwangen University, Furtwangen, Germany; 2 College of Science, Health, Engineering and Education (SHEE), Discipline of Nursing, Murdoch University, Perth, Australia; 3 Faculty of Medicine, Institute of Nursing Science, University of Freiburg, Freiburg, Germany

**Keywords:** Virtual reality, Nursing education, Skills training, Simulation

## Abstract

**Background:**

Clinical skills training is an essential component of nursing education. However, sometimes education does not sufficiently prepare nurses for the real world. Virtual reality (VR) is an innovative method to complement existing learning strategies, yet few studies investigate its effectiveness. This study compared educational outcomes achieved by three groups learning with either of two different VR simulation variants, with varying technological features, or a video training on the endotracheal suctioning skill.

**Methods:**

The investigated outcomes were knowledge and skill acquisition, learner satisfaction, and technology acceptance. 131 undergraduate nursing students were randomised into three groups, based on the interventions they received. Knowledge was assessed through a pre-post-test design, skill through a post-intervention objective structured clinical examination on a manikin, learning satisfaction and technology acceptance through standardised questionnaires, and qualitative feedback through focus groups.

**Results:**

All interventions led to a significant knowledge acquisition, with no significant difference between the groups. The video intervention group performed significantly better than the VR groups in skill demonstration. One of the two VR intervention groups had a significantly higher learner satisfaction than the video group. Technology acceptance was high for both VR groups, with the simpler VR simulation resulting in higher technology acceptance than the one with more experimental features. Students described the VR experience as realistic, interactive, and immersive, and saw the opportunity to practise skills in a safe environment, learn from mistakes, and increase knowledge and confidence.

**Conclusions:**

For the development of VR trainings, we recommend keeping them simple and targeting a specific educational outcome since trying to optimise for multiple outcomes is resource intensive and hard to achieve. Psychomotor skills were easier for participants to learn by watching a video on the procedure rather than practically learning it with the VR hardware, which is a more abstract representation of reality. We therefore recommend using VR as a complementing resource to skills labs, rather than replacing existing learning strategies. Perhaps VR is not ideal for practising practical psychomotor skills at the moment, but it can increase knowledge, satisfaction, motivation, confidence and prepare for further practical training.

**Trial registration:**

Not applicable.

## Background

### Motivation

Clinical skills are a substantial part of nursing education and nursing practice. With rapid advances in healthcare and medical research, as well as technology, students must adapt quickly and learn clinical skills efficiently [1]. However, education does not always adequately prepare students for these requirements, resulting in “weak” practical skills [2], or a theory-practice-gap [3]. To counter this deficit, several learning methods and teaching strategies, including digital, online, simulation-based, and virtual environments, have been proposed in recent years [4]. Simulation-based learning with high- and low-fidelity manikins in skills labs is a widely used and recognised strategy, which has been validated to some extent [5]. Virtual reality (VR) simulations are a new learning strategy that needs to be researched further.

### VR simulation in nursing education

On a basic level, VR can be defined as a computer-generated three-dimensional interactive environment [6–8]. Other definitions add that specialised eye- and ear-wear is required to experience it [6, 9], which is referred to as immersive VR (iVR) in newer publications [10, 11]. Virtual reality provides a high-potential opportunity for acquiring clinical skills. Learners have the chance to practise in a safe environment and learn from mistakes without harming real patients [12]. They can repeat the training easily and prepare for real clinical interventions without a teacher [13]. The capability of VR to immerse users into a simulated environment can generate a sense of presence, an emotional reaction that creates the illusion of ‘being there’, although physically in a different location [14]. Immersion and presence provide the opportunity to effectively simulate dangerous or difficult to replicate real-world situations in a virtual environment. Furthermore, VR is not location- or time-bound unlike other learning methods [13].

However, empirical evidence on the effectiveness of VR simulations in nursing education is limited [15–17]. A systematic review about VR simulation skills training for healthcare education concludes that the body of evidence is limited, and the methodological quality of studies is of ‘variable quality’ [17]. To justify the costs of investing in VR, therefore, further studies on educational outcomes such as knowledge and skill acquisition are needed [17]. Two meta-analyses – one including 9 studies [16] and the other 12 studies [15] – investigated the effects of the use of VR simulations in nursing education on learning. The first analysis concluded that VR can improve cognitive and psychomotor performance, but not to what extent [16]. The second revealed that VR was more effective than control methods in enhancing knowledge, but not skills [15]. However, these findings should be met with caution, given that both non-immersive and immersive simulations were present in the pool of studies examined. Several articles criticise that studies often do not distinguish between regular computer simulation or virtual patients (on a 2D device) and immersive virtual reality simulations (with a head-mounted display) [6, 17–19]. Some have even argued that the approach of such reviews can be fundamentally flawed, as they only aggregate effects from a technological point-of-view, while not taking into account that different VR simulations can be highly varied in their didactic design, and so their effects on outcomes are hardly comparable [20, 21]. Research on how to design VR simulations for effective outcomes in nursing education is also limited as most studies do not cover this topic at all [12, 19].

This study seeks to contribute to the literature by comparing the effects of two varying virtual reality (VR) simulations and a video training on educational outcomes, including knowledge, skills and learner satisfaction. Subsequently, it draws conclusions on how the existing technology can be utilized in nursing education; with a further exploration on what to expect from it, and how to optimise VR simulations for better educational outcomes in the future. These insights can be of aid for other researchers to effectively use VR or make upgrades to their own VR simulations.

### Endotracheal suctioning

**Fig. 1:**
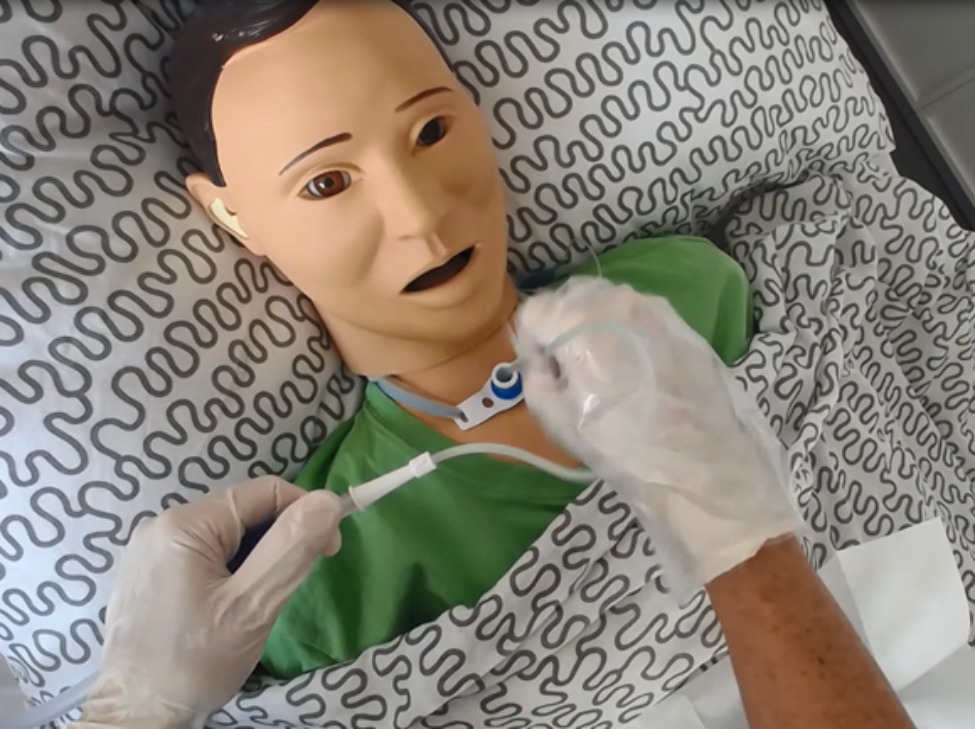
Endotracheal suctioning (ETS)

Endotracheal suctioning (ETS) is a complex skill that has been described as particularly burdensome by nurses and patients in Germany [22]. Endotracheal suctioning is performed on ventilated patients and/or those with a tracheostoma to clear the airway of secretions (see Fig. 1). Crucial aspects are aseptic non-touch techniques, and time. In general, there are standardised procedures to follow for practitioners when conducting ETS [23]. VR simulation offers an opportunity to learn such procedural skills [19].

### Aim and hypotheses

The aim of the study was to investigate the effects on educational outcomes through different learning modalities for undergraduate nursing students learning the ETS procedure. The interventions included two immersive VR simulation versions with different technical features and an educational video. The assessed educational outcomes were knowledge acquisition, skill performance, learning satisfaction, and technology acceptance. Assessment was performed quantitatively in a randomised pre-post-test design with three groups and through questionnaires to determine if there were changes that could be attributed to the type of educational intervention the participants underwent. Furthermore, complementing insights were gained through a qualitative approach. We postulated the following hypotheses:

#### H1

Participants acquire a significant increase of knowledge through either of the VR trainings or the video training.

#### H2

Participants’ knowledge acquisition differs significantly depending on the educational intervention they completed.

#### H3

Participants’ retention of knowledge differs significantly depending on the educational intervention they completed.

#### H4

Participants’ skill performance differs significantly depending on the educational intervention they completed.

#### H5

Participants’ cognitive learning satisfaction differs significantly depending on the educational intervention they completed.

#### H6

Participants’ technology acceptance’s predictor variables [a-e] regarding either the low or high VR variants differ significantly depending on which one they completed.

- *perceived usefulness*
- *perceived ease of use*
- *perceived enjoyment*
- *Attitude towards using*.
- *Intention to use*.

## Methods

### Study design

A mixed methods randomised parallel study design with three groups was proposed: Group_Video_, whose intervention was a video tutorial; Group_VRlow_, who participated in a VR intervention with basic VR technology features; and Group_VRhigh_, who received a VR intervention with more experimental technological features. Participants were assigned randomly to the three groups. Assessment comprised a knowledge test, a skill demonstration through an objective structured clinical examination (OSCE), and several questionnaires. In addition to the quantitative approach, a qualitative data collection was performed through focus groups.

**Fig. 2:**
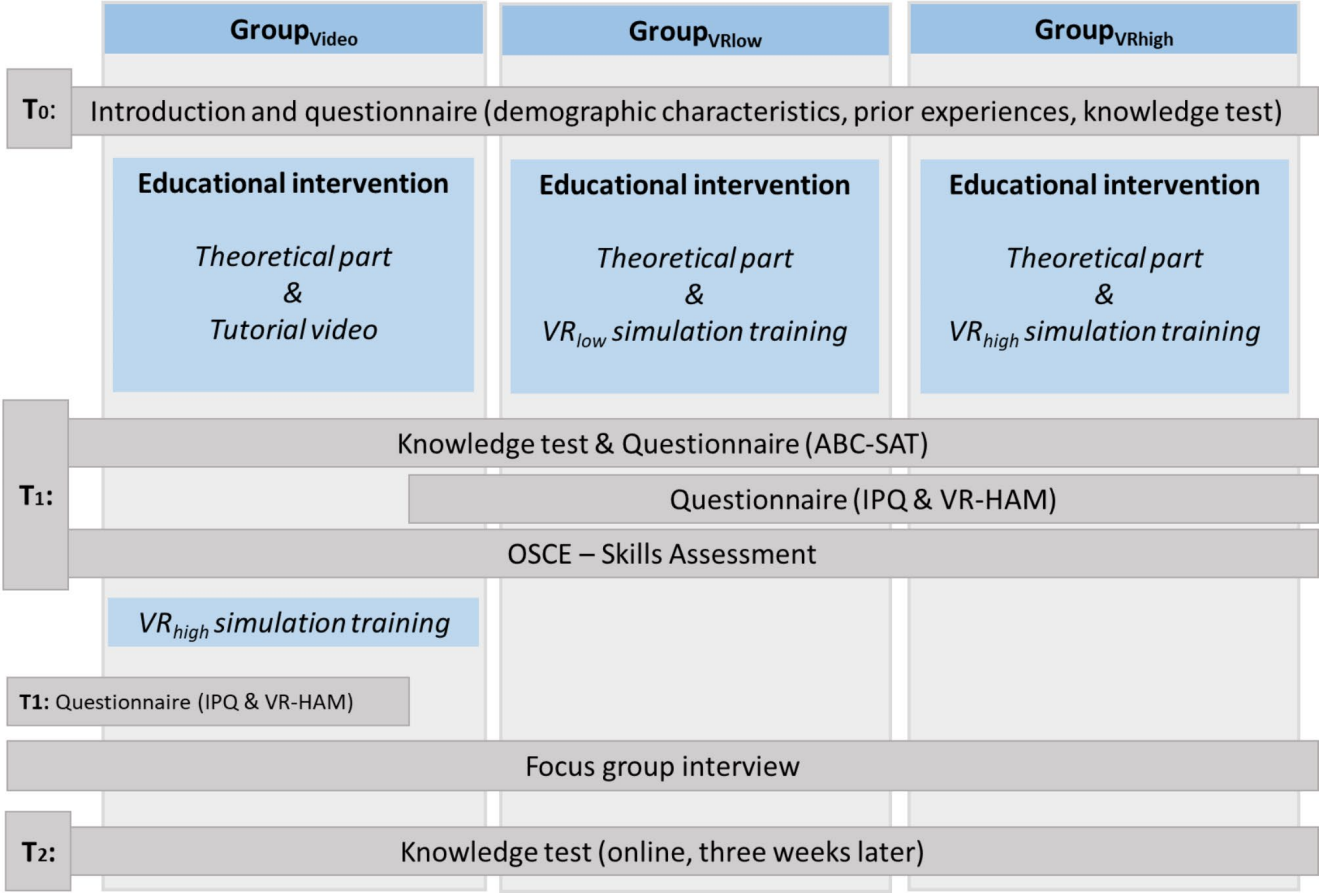
Study design. Abbreviations: ETS = endotracheal suctioning; ABC-SAT = affective- behavioural-cognitive-satisfaction questionnaire; IPQ = Igroup Presence Questionnaire; VR-HAM = virtual reality hardware acceptance model; OSCE = objective structured clinical examination

The undergraduate nursing students took part in the study intervention as part of their regular education. The whole process took two to three hours for the participants. Starting at T_0_, immediately before the intervention, participants reported demographic variables, prior experience with ETS, and did the first knowledge test. This was followed by a theoretical part with a short, recorded presentation that described the theoretical background of ETS, including the 14 steps of the suctioning process. Immediately after the interventions at T_1_, further questionnaires and the second knowledge test were carried. For fairness reasons, Group_Video_ were also given the opportunity to take part in the VRhigh intervention after completing the relevant assessment and subsequently reported technology acceptance as well. Three weeks later at T_2_, the groups filled in the knowledge test for the third time to assess retention. The whole process for the different groups and respective interventions and assessments is illustrated in Fig. 2.

### Randomisation and blinding

Randomisation was performed using the software Research Randomizer (randomizer.org) with block-wise randomisation. Based on the randomisation, participants were assigned a group and invited to participate in the study in a specific time frame according to a pre-determined schedule. No different groups were invited at the same time to prevent allocation failure. Participants were partly blinded, since they were only assigned a number and were not aware of other groups. For logistical reasons, researchers were not blinded.

### Participants and settings

In Aksoy’s recent study [24], the difference in knowledge acquisition between tablet-based and VR healthcare trainining was examined. Based on the given means and standard deviations of the respective experimental conditions, an effect size of Cohen’s d = 0.766 was assumed. The calculation of the sample size was carried out with the software G * Power 3 [25]. For the calculation of the sample size in the present study, Cohen’s d had to be transformed into Cohen’s f using the formula f = d/2). This resulted in Cohen’s f = 0.383. With an assumed effect size of f = 0.383, assuming normal distribution of the data and a predefined alpha level of a = 0.05 for the present study, a total sample size of N = 111 persons results. Assuming normal distribution, the sample calculation was exemplarily carried out based on a parametric test procedure for the comparison of three independent means.

**Fig. 3:**
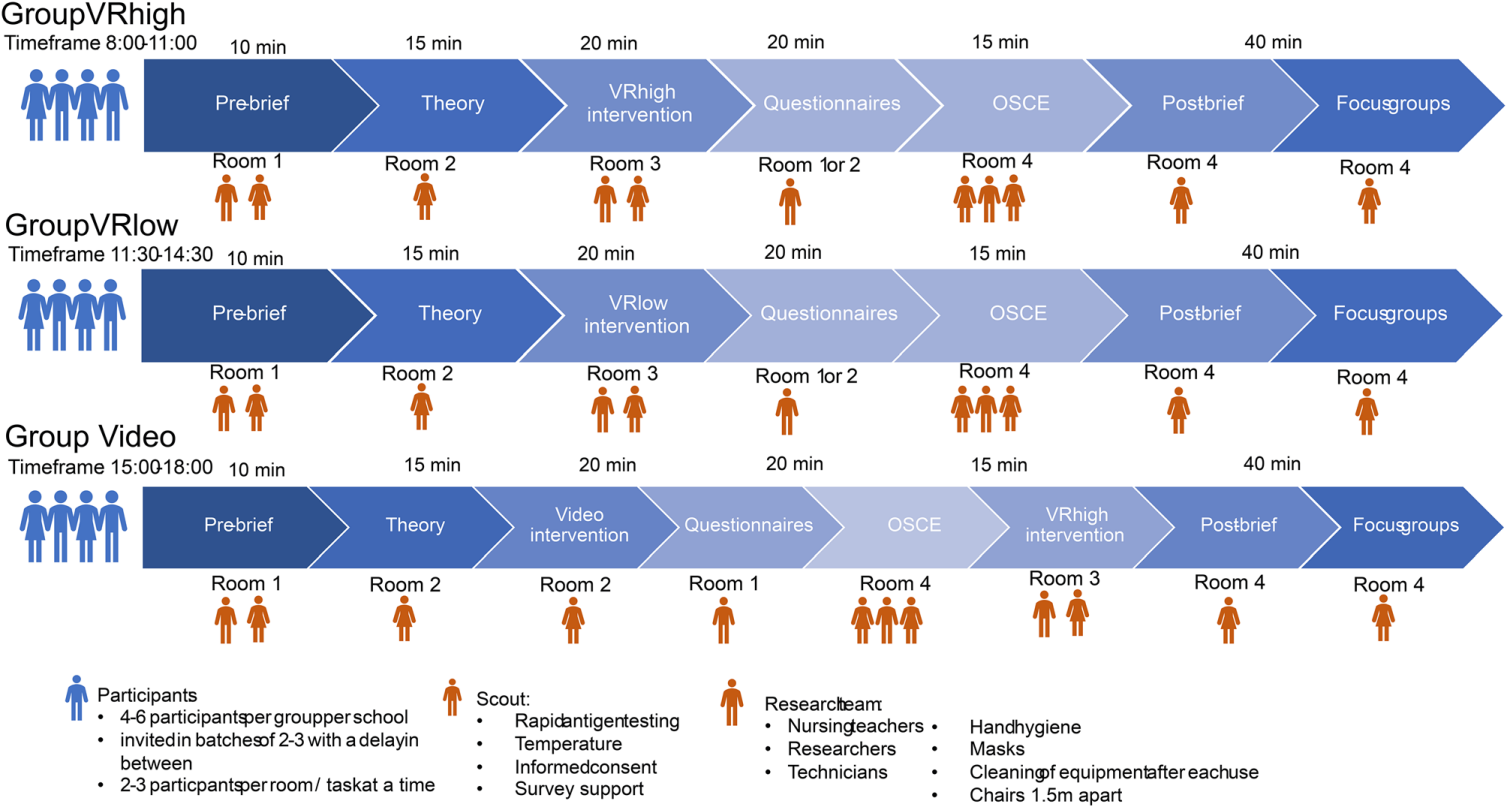
Logistics of data collection – exemplary setup of logistics for one school

The selected target group was recruited from nine nursing and anaesthetics technologist schools in the south of Germany. Second- and third-year students were informed about the study by their teachers and asked to participate in the learning activities within their curriculum. Participants were assigned to one of the three groups, with numbers ranging from four to eight trainees depending on the sizes of their classes. Up to three groups completed their interventions consecutively throughout the day. The research team comprised up to six persons, both nurses and technicians. All materials were supplied by the project team, including manikins for the OSCEs and hygiene items. Figure 3 illustrates the organisation and sequence of one intervention group.

For each participating school, the research team invited different groups at different times in order to avoid confusion and allocation problems. The setup comprised up to six pre-trained researchers with standardised operating procedures and up to four different rooms depending on group size and room availability. During the process, participants would move between the rooms depending on their next task. The order in which participants completed tasks and interventions varied depending on their group (see Figs. 2 and 3). In Room 1, pre-briefing and questionnaires took place. A nursing teacher gave the pre-briefing and a scout, who was familiar with the research, answered questions concerning the questionnaires. In Room 2, a theory presentation on the basics of ETS and the video intervention (only for Group_Video_) were presented by a nursing teacher. In Room 3, participants conducted either of the VR interventions including a VR tutorial, with two researchers providing a standardised introduction to VR and helping with predetermined cues if participants could not progress in the simulations. Back in Room 1 or 2 depending on availability, questionnaires were conducted. Finally, in Room 4, participants would individually perform the learned ETS skill on manikins as part of the OSCE observed by two to three nursing experts under a standardised protocol. Once complete, post-briefing and focus groups took place in this room, moderated by a nursing teacher and researcher experienced in qualitative methods. As the research took place at different schools on different dates, the research team varied. All researchers received a training in advance and followed standardised procedures to reduce the risk of bias. The invitation order of groups was randomly selected for each school.

### Interventions

Development of the learning package, including the VR simulations, was based on the research team’s prior experience from prototype evaluation [26, 27] and findings from our review about VR simulations in nursing education [19]. As the proposed VR training is a clinical simulation, the design aligned with the International Nursing Association for Clinical Simulation and Learning (INACSL) simulation guidelines. Their Standards of Best Practice recommend a pre-briefing of the participants for orientation and instruction, the listing of learning objectives, a safe and trustworthy environment, and a debriefing at the end of the simulation [9]. During the debriefing there was also an opportunity to ask content-related questions, which was not allowed before, in order to make the intervention as standardised as possible. In order to evaluate the feasibility of the interventions and assessment tools, a pilot study was carried out in the run-up [27], after which adjustments to the VR simulations, video, and assessment tools were made accordingly.

During the VR interventions (VRlow and VRhigh), participants had to actively perform ETS on a virtual patient in a virtual hospital room while being guided by audio instructions and visual hints. Users performed ETS step by step, with a total of 14 steps from hand hygiene to discarding used equipment. The procedure had to be executed in sterile fashion with green dots representing microorganisms upon contamination as feedback. Prior to performing ETS, users went through a tutorial to familiarize themselves with the controls and get comfortable in the immersive VR setting. Overall, participants were exposed to Virtual Reality (VR) for 20 min, comprising the tutorial for familiarization and either of the two ETS simulations. For comparison of educational outcomes, a video in which ETS is performed on a manikin by an expert, with the same explanations, audio, and visual hints, and sequence of steps was developed. More complex steps are shown from a first-person perspective. The video was chosen as a comparison learning modality to VR since it has been shown to deliver promising results in psychomotor skills training [28].

**Fig. 4:**
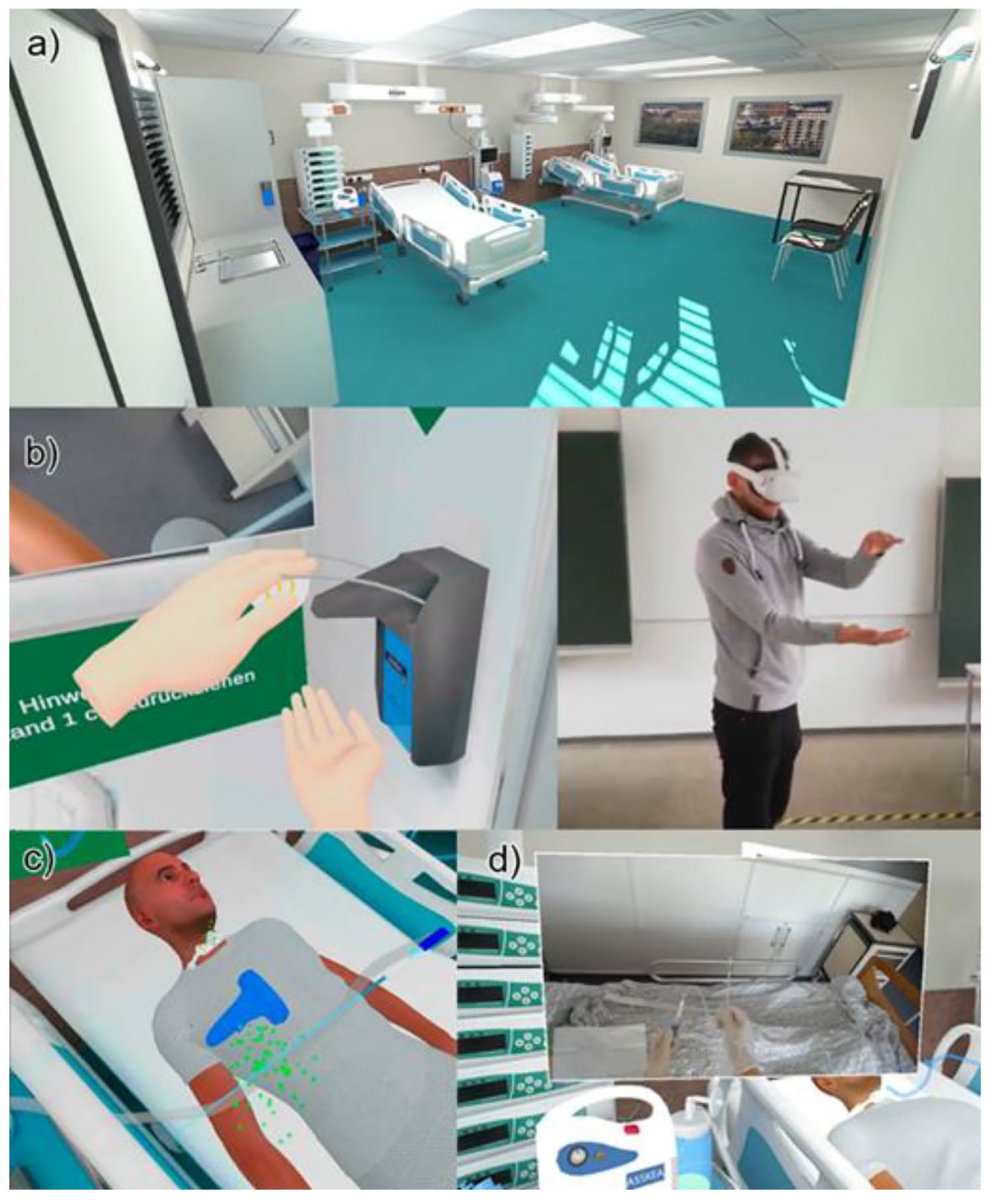
Image collage on the VR simulation features. **a**) the setting and graphics that participants could see through the VR simulations that is the same in both versions; **b**) the hand-tracking feature that is only present in the VRhigh simulation whereas the VRlow simulation uses standard Oculus Quest 2 controllers; **c**) visualisation of microorganisms to illustrate hygiene errors present in both versions; **d**) in-situ video clips for demonstration of psychomotor skills in the VRhigh simulation. Figure based on [27]

The VRlow and VRhigh versions derive from the same simulator, so the procedure of steps that had be executed, the graphical detail and most other features are equal. There are however two major differences: The VRlow version features a controller-based input and tracking system, similar to a video game, while VRhigh incorporates the Oculus™ hand-tracking and input system, enabling users to see and interact with their actual hands. In addition, VRhigh was supplemented with video-clips from the video intervention, providing an additional layer of information in VR on how to perform more advanced psychomotor skills such as unpacking a suction catheter or donning a sterile glove. The differences and similarities of the two versions can be viewed in Table 1, and Fig. 4.

**Table 1:** VRlow and VRhigh interventions

Interventions	VRlow	VRhigh
Visual and textual hints	Yes	Yes
Audio guiding	Yes	Yes
Device	Oculus Quest 2™	Oculus Quest 2™
Input	Controllers	Hand gestures
Tracking	Head and controllers	Head and hands
Real-world video clips	No	Yes

### Outcome assessment

A summary of the assessment tools is outlined in Table 2 below. All questionnaires were set up online in a LimeSurvey™ and accessed via smart devices and QR codes but could be filled in as paper versions if there was no internet available.

**Table 2:** Outcomes and assessment tools

Outcome	Assessment tool	Timing
Increase of knowledge re ETS	Knowledge test	before (T_0_) and after intervention (T_1_); after 3 weeks (T_2_)
Practical skill	OSCE	T_1_ after any learning intervention
Satisfaction with learning	ABC-SAT	T_1_ after any learning intervention
Acceptance of VR technology	VR-HAM	T_1_ after a VR intervention
Qualitative data	Focus groups	T_1_ after quantitative assessment

Acquisition and retention of knowledge was assessed by a self-developed knowledge test consisting of eight multiple-choice items (each with four possible answer options) and two further items where the individual steps of conducting ETS had to be placed into the correct order. The maximum score was 10. The knowledge tests were made by a nursing teacher and researcher and reviewed by another.

To assess the degree of acquired skills, participants conducted an OSCE after the intervention, where the learned skill was demonstrated on a manikin and rated by an expert observer. A maximum of 14 points could be achieved for a flawless execution of the procedure with steps in the correct order and no errors, such as contaminations or leaving the patient off the ventilator for too long.

Both, the knowledge tests and the OSCEs were performed by participants individually without cooperation.

Participants’ satisfaction with the educational intervention was evaluated by the affective- behavioural-cognitive-satisfaction questionnaire (ABC-SAT) [29]. The questionnaire has 11 items, divided into the three subscales: affective, behavioural, and cognitive satisfaction. Participants rate their level of agreement on a 5-point Likert scale (from 0 = *does not apply at all* to 4 = *fully applies*). Only the six items from the cognitive satisfaction subscale were considered relevant in the context of our study and were included. The score of this subscale ranges from 0 to 24, whereby a higher score implies higher satisfaction.

To measure the acceptance of the used VR technology, an adapted version of the virtual reality hardware acceptance model (VR-HAM) was used [30]. Participants had to respond to 23 items from five subscales (*Perceived usefulness*, *Perceived ease of use*, *Perceived enjoyment*, *Intention to use*, and *Attitude towards using VR hardware*) by stating their level of agreement on a 5-point Likert scale (from 1 = *strongly disagree* to 5 = *strongly agree*).

The number of participants in the qualitative results differed from the quantitative, since there was an additional group participating in both video and VR training, which was excluded from the quantitative analysis since it would not have been adjusted to the power calculation and the selected statistical methods. However, their qualitative feedback could still include important information. Therefore, for the qualitative analysis, n = 170 students, as well as some teachers, participated in 15 focus groups (duration between 9 and 21 min) with open-ended questions. The focus groups were audio-recorded and then transcribed. Data was analysed with MAXQDA using the systematic qualitative text analysis approach by Kuckartz [31], a descriptive analysis method that focuses on the formation of thematic categories.

In a first phase, the statements were coded in the main categories formed deductively from the interview guide. During the second stage of the evaluation, the inductively formed subcategories as well as further main categories were identified, and the entire transcription material was finally coded and evaluated. By comparing and contrasting the text passages in the individual categories, a particularly good level of differentiation, complexity, and explanatory power is achieved in this type of category-based evaluation and presentation [31]. The qualitative results chapter is structured by the main categories that emerged:

### Statistical evaluation

All data from paper versions of the questionnaires was entered into the LimeSurvey™ and downloaded for analysis. Statistical analysis and evaluation were performed in SPSS™. Significance of differences between groups for the time-dependent variables *increase* and *retention of knowledge* were analysed with a mixed analysis of variance (ANOVA). Differences between groups for other outcomes with only one measurement were evaluated with either a one-way ANOVA or a Welch-ANOVA, depending on homogeneity of variances. Accordingly, for post-hoc analyses we used Tukey’s honest significant difference (HSD) test or the Games-Howell test. For analyses comparing only the two interventions VRlow and VRhigh, independent t-tests were conducted. Alpha level was set at α = 0.05. Cohen’s d served as an effect size measure to compare group differences. Analyses for each construct were run separately with subjects being excluded from it if relevant data was missing.

## Results

### Sample

The sample consisted of n = 131 nursing students and one drop-out due to severe cybersickness. 119 reported their age between 18 and 54 (M = 23.24, Mdn = 21, SD = 5.88). The sample consisted of 74% female and 26% male participants reflecting the gender balance in the field. Due to logistical reasons, there were slight imbalances in group sizes with Group_VRhigh_ (N = 47), Group_Video_ (N = 43) and Group_VRlow_ (N = 41). 53% of participants had previous theory knowledge on endotracheal suctioning through education and 65% had practical know-how through previously conducting or observing the procedure. In contrast, only 32% had previous experience with VR and only 5% had personal VR equipment at home. While around 50% suffered from migraine, only 8% were affected by travel sickness. There were no significant differences in previous experience with VR or ETS between the groups. Table 3 shows the descriptive attributes overall in detail while Table 4 shows the distribution for each group.

**Table 3:** Independent variable distribution among all participants

Attributes	yes	no
Prior experiences with VR headsets	42 (32.0%)	89 (67.9%)
VR headset at home	5 (3.8%)	126 (96.2%)
Prior theory knowledge about endotracheal suctioning	70 (53.4%)	61 (46.6%)
Practical experience with endotracheal suctioning	86 (65.7%)	45 (34.3%)
Affected by migraine headache	65 (49.6%)	66 (50.4%)
Affected by travel sickness	8 (6.1%)	123 (93.9%)

**Table 4:** Independent variable distribution among groups

Group	Video		VRlow		VRhigh	
Answers (yes/no)	yes	no	yes	no	yes	no
Prior experiences with VR headsets	14 (32.6%)	29 (67.4%)	12 (29.3%)	29 (70.7%)	16 (34.0%)	31 (66.0%)
VR headset at home	2 (4.6%)	41 (95.4%)	2 (4.9%)	39 (95.1%)	1 (2.1%)	46 (97.9%)
Prior theory knowledge about endotracheal suctioning	21 (48.8%)	22 (51.2%)	21 (51.2%)	20 (48.8%)	28 (59.6%)	19 (40.4%)
Practical experience with endotracheal suctioning	26 (60.5%)	17 (39.5%)	25 (61.0%)	16 (39.0%)	35 (74.5%)	12 (25.5%)
Affected by migraine headache	22 (51.2%)	21 (48.8%)	21 (51.2%)	20 (48.8%)	22 (46.8%)	25 (53.2%)
Affected by travel sickness	1 (2.3%)	42 (97.7%)	1 (2.4%)	40 (97.6%)	6 (12.8%)	41 (87.2%)

### Knowledge acquisition

Table 5 shows a summary of scores that were achieved by the different groups in the equivalent pre- and post-knowledge tests that took place at a point in time immediately before (T_0_) and immediately after (T_1_) each group’s intervention. In total, groups achieved a mean score of 4.83 in the T_0_ test and 7.14 in the T_1_ test. Every group had a highly positive score increase (M_T1_ – M_T0_) and increased their score in T_1_ by a mean between 2.18 and 2.39 compared to T_0_, representing a relative change between 43% and 50% in reference to their initial score in T_0_.

**Table 5:** T_0_ and T_1_ knowledge test results

Group	Knowledge test	M	SD
Video	T_0_	4.77	1.41
	T_1_	7.16	2.29
VRlow	T_0_	5.02	1.46
	T_1_	7.20	1.44
VRhigh	T_0_	4.72	1.29
	T_1_	7.06	1.42

A mixed ANOVA determined how meaningful the differences in scores were between groups and within points in time. The variable *group* (Video, VRlow, VRhigh) was used as between-subject (BS) factor, and the *point in time* (T_0_, T_1_) at which the tests took place as within-subject (WS) factor, with the test score achieved as the dependent variable. There was no significant main effect for group membership (*F*_*BS*_ *(2, 128) = 0.31, p = .730, partial η² = 0.005*), meaning that intervention groups did not differ significantly regarding outcomes in knowledge tests. There was a significant main effect for time (*F*_*WS*_ *(1, 128) = 205.98, p < .001, partial η² = 0.616*), confirming that there was a significant increase in knowledge test scores between T_0_ and T_1_. Lastly, there was no significant interaction effect between BS and WS factors (*F*_*BS×WS*_ *= (2, 128) = 0.17, p = .844, partial η² = 0.003*), which means that group membership had no effect on knowledge acquisition between T_0_ and T_1_. In conclusion, every group’s intervention led to a significant knowledge acquisition, confirming H1, but acquisition did not differ between groups, rejecting H2.

A summary of scores achieved by the groups at T_0_, T_1_, and at T_2_, three weeks after the intervention in a third equivalent knowledge test are outlined in Table 6. Out of the N = 131 participants, n = 53 38.9%) returned a knowledge test for T_2_. Therefore, the following analysis is conducted with a subsample, and results at T_0_ and T_1_ differ from the above analysis of the larger sample. Scores achieved at T_2_ slightly decreased compared to T_1_, with a score decrease between 0.06 and 0.57 points (M_T2_ - M_T1_) depending on the group. The relative change in reference to the previous increase, meaning the percentage of previously gained knowledge between T0 and T1 that was lost between T1 and T2, varies between 3% and 24%.

**Table 6:** T_0_, T_1_ and T_2_ knowledge test results

Group	Knowledge test	M	SD
Video	T0	5.35	1.50
	T1	8.12	1.17
	T2	7.82	0.88
VRlow	T0	5.05	1.40
	T1	7.43	1.40
	T2	6.86	1.11
VRhigh	T0	5.40	1.35
	T1	7.73	0.80
	T2	7.67	1.40

A mixed ANOVA including the additional point in time (T_2_) in the WS factor was conducted in a similar fashion as described for the T_0_-T_1_ analysis. Likewise, there was no significant main effect for group membership (*F*_*BS*_ *(2, 50) = 2.41, p = .100, partial η² = 0.088).* There was a significant main effect for time (*F*_*WS*_ *(2, 100) = 99.55, p < .001, partial η² = 0.666*), meaning knowledge changed significantly between points in time. There was no significant interaction effect between group membership and time (*F*_*BSxWS*_ *(4, 100) = 0.74, p = .570, partial η² = 0.029*), which means group membership did not affect knowledge change between points in time. It can be concluded that there was a significant increase in knowledge between T_0_ and T_1_, and a significant retention between T_1_ and T_2_. Acquisition and retention did not significantly differ between groups. Therefore, H3 can be rejected.

### Skill demonstration (OSCE)

**Fig. 5:**
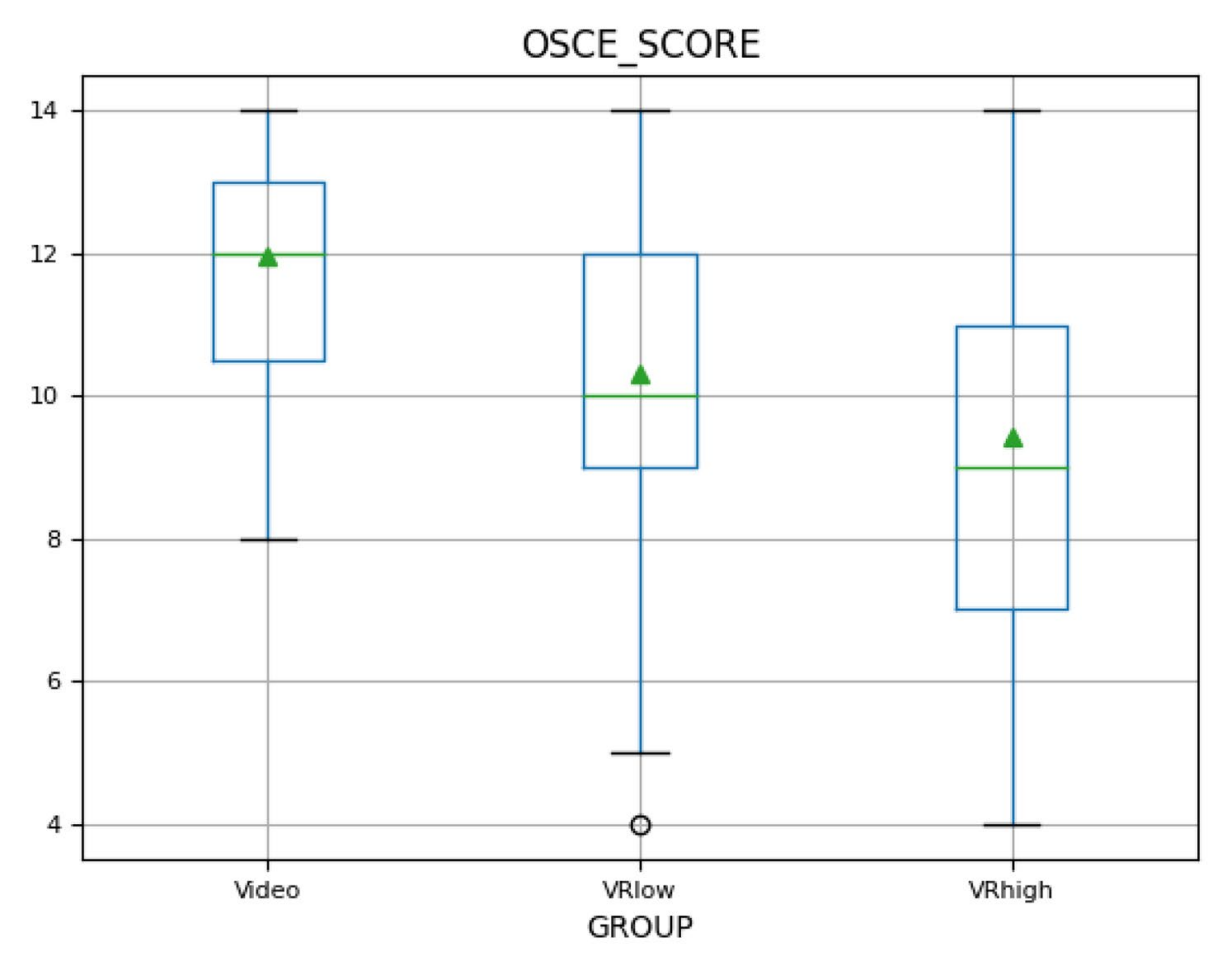
OSCE results boxplot. Boxplot notation: green triangle = mean; green line = median; blue box = 25th to 75th percentile; black lines = 0th to 100th percentile excluding outliers; black circles = outliers

Compared to narrower performance gaps between groups in knowledge tests, skill competency assessed through OSCEs immediately after the group’s interventions was more broadly distributed with mean scores varying between 9.41 and 11.95 out of a maximum of 14 points. Group_Video_ achieved the highest score (M = 11.95, SD = 1.65), followed by Group_VRlow_ (M = 10.32, SD = 2.22), and finally Group_VRhigh_ (M = 9.41, SD = 2.70), as illustrated in detail by Fig. 5.

Since Levene’s test indicated non-homogenous data, a Welch-ANOVA was conducted, revealing that differences between groups were significant (*F (2, 81.63)* = 16.97, *p* < .001), confirming H4. A Games-Howell post-hoc test gave insight into which intervention groups performed significantly better than others in the OSCE. Group_Video_ performed significantly better than both, Group_VRhigh_ (*d = 1.15, p < .001*) and Group_VRlow_ (*d = 0.83, p < .001*). The results illustrate that the VR groups, especially Group_VRhigh_, were less successful in demonstrating the learned skill on a manikin. The range of achieved scores was higher for the VR groups, especially Group_VRhigh_ as can be seen in the boxplot (see Fig. 5).

### Learner satisfaction (adapted ABC-SAT)

The cognitive learning satisfaction scores varied between means of 16.9 and 19.93 out of a maximum of 24, with Group_VRlow_ reporting the highest cognitive satisfaction (M = 19.93, SD = 4.60), followed by Group_VRhigh_ (M = 17.96, SD = 5.74), and then Group_Video_ (M = 15.72, SD = 7.20). The scores illustrate that participants were cognitively satisfied to very satisfied with the VR learning interventions.

**Fig. 6:**
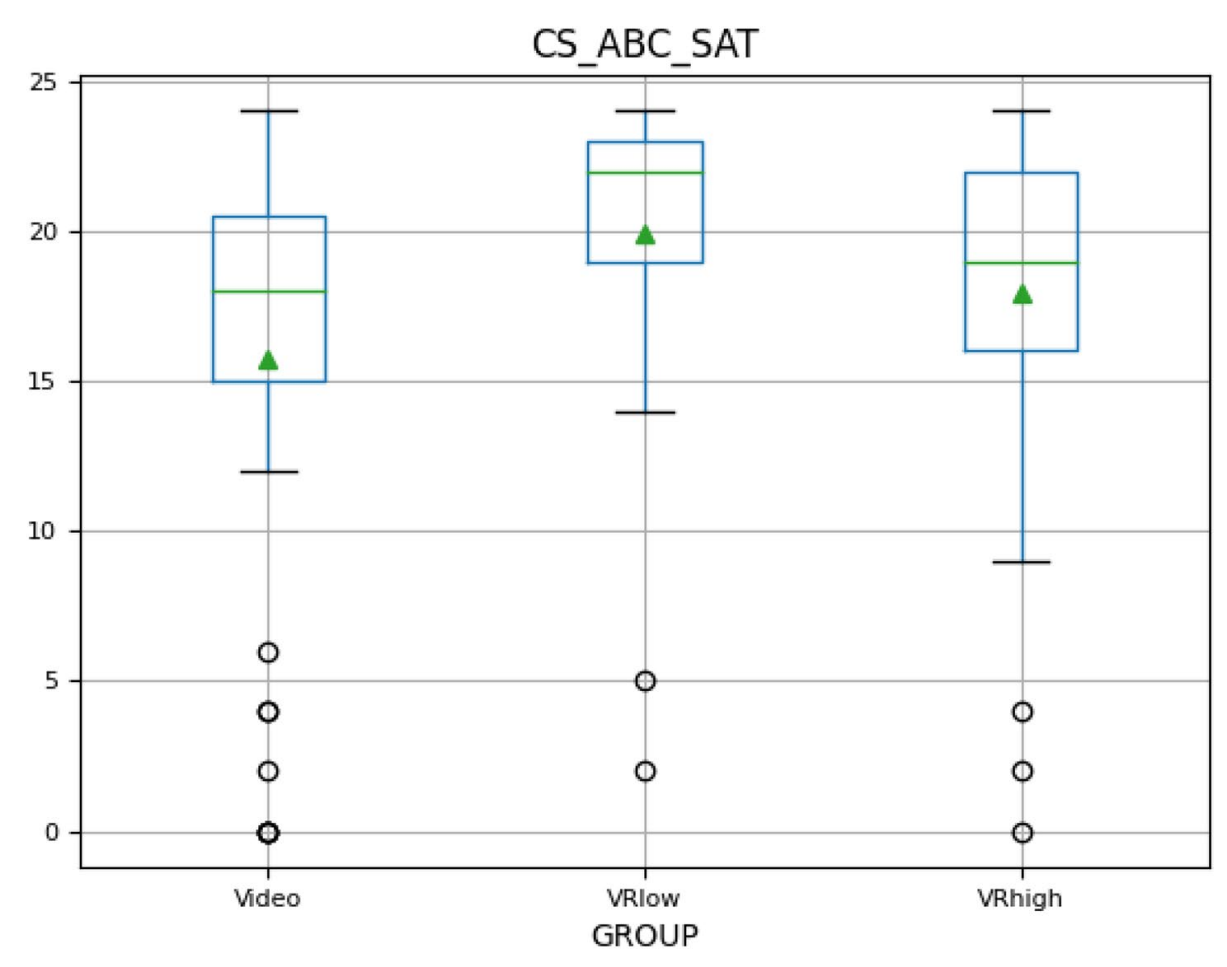
Cognitive satisfaction boxplot

The boxplot (see Fig. 6) visualises the range of reported satisfaction scores with relatively many outliers. A one-way ANOVA indicated significant differences between the groups (*F = 5.26, p = .006*), confirming H5. A Tukey-HSD post-hoc test indicated that Group_VRlow_ was significantly more satisfied than Group_Video_ (*d = 0.70, p = .004)*.

### Acceptance (adapted VR-HAM)

As described in the study design section, all three groups eventually received either the VRlow or VRhigh intervention prior to reporting technology acceptance through the VR-HAM. Therefore, for this analysis, we grouped participants into VRlow and VRhigh recipients only, regardless of their intervention group. Since Group_Video_ received the VRhigh intervention after group-specific assessment, the VRhigh recipient count (N = 87) was significantly higher than VRlow (N = 41). As shown in Table 7, the means of *Perceived usefulness*, *Perceived ease of use*, *Perceived enjoyment*, and *Attitude towards using* were on the upper end of the Likert scale, reaching values around 4 out of 5 points indicating a positive attitude and positive expectations towards the technology in the sample. Actual *Intention to use* was slightly lower, with values close to 3. VRlow recipients reported slightly higher values in means across all constructs.

**Table 7:** Acceptance of VR technology results

VR-HAM construct	Intervention	M	SD
a) Perceived usefulness	VRlow	4.01	0.79
	VRhigh	3.79	0.87
b) Perceived ease of use	VRlow	3.96	0.55
	VRhigh	3.58	0.64
c) Perceived enjoyment	VRlow	4.44	0.52
	VRhigh	4.14	0.81
d) Attitude towards using	VRlow	4.26	0.58
	VRhigh	4.01	0.73
e) Intention to use	VRlow	3.26	0.94
	VRhigh	3.03	0.81

Independent t-tests with Cohen’s d as an effect size measure were performed to investigate differences in the construct means. VRlow recipients reported significantly higher values for *Perceived ease of use* (d = 0.62, p = .002), *Perceived enjoyment* (*d = 0.40, p = .037*). *Perceived usefulness* (d = 0.36, p = .057) and *Attitude towards using* (d = 0.37, p = .057) were close to being significantly different, while Intention to use was not significantly different. Therefore, H6b and H6c can be confirmed, while no conclusive statement about H6a and H6d can be made, and H6e can be rejected.

### Qualitative results

#### Experiencing VR

Learning with VR was a lot of fun for almost all respondents. It was described as amusing, (mega-) cool, exciting, great, a bit confusing, also hilarious and something totally new. It was easy to get immersed in the virtual world and to get used to it quickly. Virtual reality was experienced as a very good medium to visualise procedures and was seen as a useful teaching method. For the trainees, VR meant stress-free learning in a *“protected”* space, *“cut off from reality”* (A1_video, position 10–10). Being protected referred to both the learner, who did not feel observed, and potential patients as this complex skill could be practised without risk or danger on a virtual person.

Newcomers experienced the learning method via VR as strange and taking time to get used to. They reported that it required a lot of concentration to adjust to the new medium, so that less attention could be paid to the teaching content. However, they conceded that this would become easier with more exposure to VR.

Many statements were made about how deeply the participants felt immersed in the VR simulation and that they had completely forgotten about reality.

*“When I put on the headset, it was such a flash that it looked like so real” (B1_low, position: 9–9). “I was very careful with the simulation on the patient, because I somehow had the feeling the whole time that he was totally aware of it.” (C1_pilot, position: 6–6)*.

#### Learning with VR

**Fig. 7:**
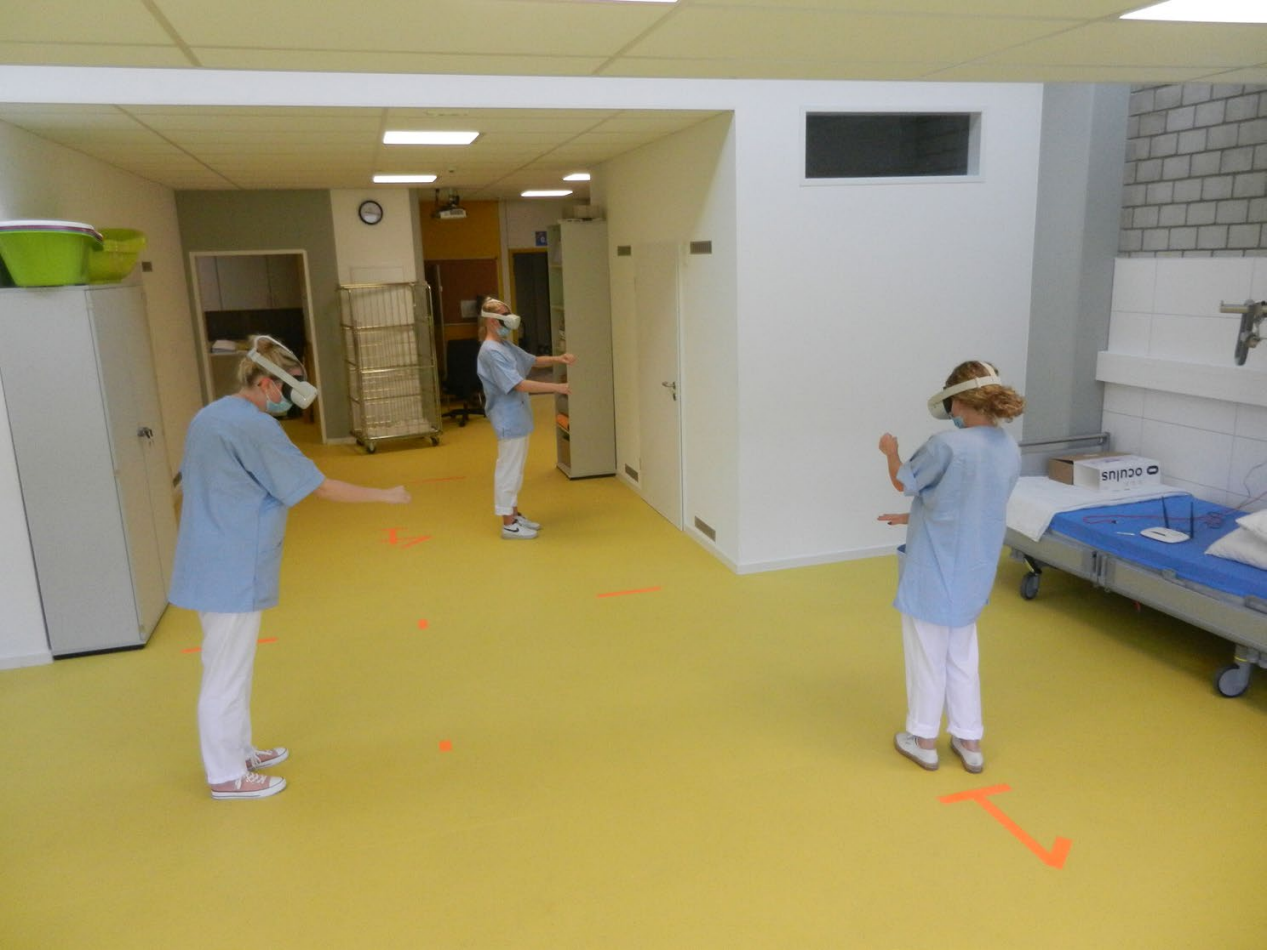
Participants during the VR simulation

The VR method made it possible to become familiar with the procedure and to repeat it multiple times during the learning process. Due to the active participation in the simulation (see Fig. 7), it was easier to remain focussed and maintain concentration. This type of learning was therefore considered much more helpful compared to, for example, a PowerPoint™ presentation or a video, in which one *“switches off at some point”* (D1_video, position: 6–16). Since the individual steps were actually carried out during the simulation, the content could be better memorized and linked to theory. The students felt that this would especially be the case if VR was integrated into a learning package with theory and practical execution on a manikin.

#### Advantages and disadvantages of VR simulations

Learning with VR meant learning with various senses: seeing, hearing, and proprioception, i.e., position in space and movement. Additionally, there is the psychomotor aspect of the task. This combination deepened the learning experience. Once purchased, VR headsets could always be available and enable learning and practising independent of an instructor or skills lab.

*“There is someone there to guide you, but I get the information also in writing. I can read it and hear it at the same time and see all the materials already there. I find that a very, very good aspect. It can always be repeated.”* (E1_video, position: 20–20).

Participants were surprised to find their way around the virtual world so easily. As it was possible to immerse oneself in a realistic world the theory-practice transfer was perceived as successful for many participants, despite the deficits in handling and haptics. They found it very helpful to be guided through the scenario by the individual steps being explained and internalised through participation.

*“I found it very easy to get the process right and to do the right steps one after the other. Better than if you had read it through beforehand, for example, then I wouldn’t have had it in my head like when I did it myself with the headset.”* (B1_low, position: 11–11).

The fact that some hand movements were not doable or realistic due to the limitations of the technology was seen as a significant disadvantage. As a result, mistakes were made which would not have occurred in reality. This led to uncertainty. Fine motor skills, which pose the greatest challenge and thus the greatest need for practice in this complex nursing procedure, could not be adequately practised. VR simulation was described “*very far away from reality in terms of handling*” (B1_video, position 10–10).

Students inexperienced with VR felt at times so distracted that they could not process all the impressions and they could not focus on the actual task. There was talk of “*too much input*” and “*overload*”, which made learning with VR almost impossible or only to a limited extent.

The dialogue with a teacher during learning was important for many trainees and was missed during the VR simulation.

*“You can’t talk there, so I miss the subjective experience. I just need someone I can talk to. I prefer that. I find it easier.”* (B1_high 14–14).

#### Practising structured nursing procedures

The learning method of VR simulation lends itself to practising clearly structured procedures, as the simulation can be carried out multiple times in succession, depending on the need for practice.

*“I think it’s a great way to prepare for practice, because I can do it 20, 30, 50 times in a row so that I have the procedure down pat. I can’t do it on the ward, for example, because I always have to hope that there is a patient there, and then I do it once, because the patient doesn’t need it more often at that moment.”* (A1_high, position: 27–27).

#### Suggestions for improvement

Most suggestions for improvement related to handling and haptics. Participants particularly reported handling problems with the suction catheter behaving rigid rather than flexible as in reality, and the lack of possibility to practise fine motor skills. They mentioned a “weird hand position” when holding the suction catheter, and that activities were triggered in the simulation that had only been initiated to some extent or not at all. Furthermore, the lack of gravity, i.e. that the catheter could not fall to the ground, was also criticized.

Participants wanted the simulation to start with the preparation of all materials rather than having everything already prepared and available. This would make the simulation more practical and increase the learning effect. The interviewees also wanted the patient to show more reactions, for example, moving his eyes, saying something, or coughing when he is being suctioned.

The trainees would like to have a simulation with immediate feedback in order to be able to correct or rehearse a specific step in the sequence promptly; a red warning light was mentioned as an idea.

*“I would have found it even cooler if they had said: ‘Okay, let’s redo mistakes’. I basically thought I did everything right and then did it like that in the practical exam.”* (B1_high, position: 8–8).

The possibility to select different difficulty levels for the simulation would be helpful for a progressive learning success. A selection option for right-handers and left-handers was also desired, as well as an option for reducing the speed of the individual activities in the overall process.

#### Environmental factors

This mainly encompassed the poor equipment of the schools regarding internet availability and computer use. It was also mentioned that teachers were often not open to new digital technologies or lacked the necessary competence.

#### Cybersickness

The respondents experienced the following symptoms of cybersickness: Headaches, which were usually mild, dizziness, nausea, and blurred vision, all of which occurred very rarely.

#### Further possible applications and limitations of VR

The trainees could imagine further applications for VR simulation for mainly standardised activities: placement of an indwelling urinary catheter, insertion of a nasogastric tube or a peripheral venous cannula, measuring blood sugar levels, intubation, resuscitation, performing simple and complex dressing changes on different parts of the body, as well as care for a ventilated patient.

#### Comparison of learning methods

During the focus groups, participants were asked to evaluate VR compared to other learning methods, e.g. watching the practical demonstration via video. However, many participants mainly compared the VR application with the practical exercise on the manikin (conducted as an OSCE in the context of the study). A very large proportion reported that they had experienced the greatest learning success through the haptics and the handling during the exercise on the manikin as well as through the repetition and the targeted individual feedback.

*“So, the VR is more fun, I would say, but I think it is more realistic on the manikin, because you can feel resistance or something, especially when suctioning. You just don’t have that with VR.*” (E1_high 4–4).

Recorded videos in VR or “only” learning videos without VR were also experienced as good learning methods, since they came closest to the real world. Many aspects of the procedure were later recognised and remembered during one’s own execution of the skill.

*“I think that the video helps us to memorise the individual steps much more quickly. Especially when you see someone demonstrating it to you, I think it’s much easier than if you’re thrown in there and have to do it alone with this VR headset. Even if you are told the steps, but I think it’s easier to remember when you see someone doing it.”* (C1_pilot, position: 26–26).

A combination of the different learning methods was considered most helpful.

*“The fine motor thing wasn’t so accurate [with VR], but that’s why I thought the combination of hands-on with the manikin and that with the headset was very good. Because with the headset you can structure the process very well and the fine motor skills can then be practised quite well on the manikin.”* (A1_video, position: 12–12).

Regardless of how great the learning success was with the different methods, the trainees wanted variety and fun in their learning, which was certainly the case with the VR simulation.

*“Well, I think if you could somehow incorporate something like that into everyday school life, it would definitely be a highlight, now compared to a dry presentation.”* (F1_video, position: 5–5).

#### Future visions

One idea was to set up a special VR room, which was almost empty except for two to four VR-headsets and the walls lined with foam to prevent injuries. Such a room would be very useful for independent learning. It should be freely accessible to trainees so that they could practise at any time.

*“Maybe even when you’re on the ward and you’re doing special care, like changing a dressing or something. And if you are unsure, you can say: ‘Okay, then I’ll go upstairs after my shift and practise again with the headset and get to know it a bit better”.* (G1_video, position: 54–54)

Virtual reality would offer many opportunities and possibilities for learning and practising at home, as well as in preparation for a clinical placement. Schools should purchase VR hardware and software so trainees could borrow them. If the VR simulations could be improved to become even closer to reality, the current deficits should be surmountable. The future will show what will be possible.

*“I think in the future it will become a really big thing.”* (B1_video, position:14–14).

## Discussion

### Reflection of results and comparison with other studies

#### Knowledge acquisition

All three groups significantly improved their theoretical knowledge. There were no significant differences between the groups, indicating that neither of the interventions had an advantage over the others. It can be concluded that both VR and videos can effectively be used to acquire knowledge for the ETS skill in nursing education.

As described in the background, different VR simulations can vary in both technology as well as didactic simulation design and making comparisons can be difficult. We tried to find similar studies to compare ours against but did not find any that compared knowledge acquisition between VR and video training in nursing education. However, like our study, [32] found no significant difference between two varying levels of immersive VR simulation regarding knowledge acquisition.

A study that compared two VR simulations for medication administration – one more active and one more passive – also saw no significant differences between the two in knowledge acquisition [33]. On the contrary, one study found significantly higher knowledge acquisition for a group with VR training compared to one with a tablet-based serious game [24]. Overall, few studies compare knowledge acquisition and skill performance achieved through different simulation designs and levels of immersion, and the compared interventions are often very different.

The distinct results of these studies reflect these circumstances. Further research comparing VR simulations to other learning methods is required in order to improve simulation design to utilise the technology more effectively. VR simulations in nursing education need to be validated and standardised through high quality studies. Only then general conclusions on the effectiveness of VR in nursing education can be made.

#### Practical skills acquisition

Practical skills acquired varied between the groups. Group_Video_ performed significantly better than both VR groups in skill demonstration. Learning the procedure by observing a real person conducting it therefore resulted in better practical skills than doing an abstraction of the procedure in our virtual environment. Insufficient fidelity and the lack haptic feedback in our simulation may have played a role in this discrepancy, as it is crucial for mastering complex psychomotor skills [34, 35]. Further details on this evaluation can be found in the section *Evaluation of the VR Simulations and Their Distinct Features.*

In addition, the commonly held assumption that increased immersion in Virtual Reality (VR) automatically leads to improved learning and skills outcomes is to be challenged [36]. In some cases, too much immersion can lead to cognitive overload and impede the learning process [36, 37]. VR simulations are complex constructs with many variables which can be adjusted through hardware, software, and didactic design.

#### Learner satisfaction and acceptance

Cognitive learner satisfaction differed significantly between the groups. Group_VRlow_ reported a significantly higher cognitive learner satisfaction than Group_Video_. The inter”active” and gamified VR learning method thus led to a higher cognitive satisfaction than the “passive” video tutorial. Overall, the high satisfaction with VR as a learning method could lead to an increased student motivation and attractiveness of nursing education.

The technology acceptance among learners was high and the target group was open-minded towards using the technology. The VRlow simulation was perceived as slightly easier and more enjoyable to use showing a slightly more positive attitude towards using it and indicating that the controller-based input system was more user friendly and intuitive than hand-tracking. Compared to other studies evaluating the acceptance of VR in the nursing education domain, we come to similar conclusions and can state that nursing students are open to learning with this novel technology and perceives a high potential [38, 39].

#### Qualitative feedback

The findings from the focus groups in our project were similar to those by [40]. Their study investigated perceptions of acceptability and applicability of an immersive VR simulation game on sepsis and found that participants described the VR experience as realistic, interactive, and immersive. Like our students, they saw the opportunity to practise in a safe environment, learn from mistakes, increase knowledge and competence – and thus confidence – but also noted the limitation of not having a “human factor” and therefore the VR simulation should be complementing a skills lab.

In their reflections, participants in a VR simulation on client-centred care stated that VR provided the opportunity to look at a situation from a new perspective and that engagement in learning was greatly enhanced and was a “refreshing change from traditional course work“ [41]. The researchers also commented on how eager and excited the nursing students were – “like kids on a Christmas morning“. Our focus groups came up with similar quotes appreciating the feeling of being immersed in a scenario and receiving feedback after completing the skill in the VR scenario.

### Evaluation of the VR simulations and their distinct features identifying pain points

#### Novelty of VR simulations

Based on the focus group feedback, we could potentially improve knowledge outcomes of our VR simulations by providing better feedback on procedural errors, for example with a replay that highlights and explains certain actions. In addition, tailoring the speed and information density in the simulation to the individual – for example by offering a choice of different difficulty settings – might also prove helpful.

There are several possible reasons why the video led to better skill performance than the VR simulation. The first reason could be the lack of previous experience with VR. 70% of participants were first-time users and most likely were too busy processing the new technology to fully focus on learning the actual procedure. Although the simulation contained a VR tutorial, it could be beneficial to let students use it repetitively over a longer period and adapt to it – especially when multiple difficulty settings are available. Some participants felt like they were “thrown into the deep end” of the VR experience. Perhaps a better pre-briefing, a more detailed explanation of ETS on what to do and what to expect in the VR simulation could help with this issue. Other participants mentioned that they could remember the steps of the procedure better when seeing a person do and explain it in a video rather than by performing the procedure by themselves in VR simulation.

#### Simulation accuracy

Group_Video_ was presented with the same procedure and setting as the OSCE, while the setting in the VR simulations deviated. The VR scenario took place in a virtual intensive care hospital environment with a virtual patient instead of a real-world simulation mannequin in a skills lab like in the video and the OSCE. The virtual equipment and materials were not accurate replicas and behaved differently compared to the real ones. Most noticeably, the VR groups did not develop fine psychomotor skills as compared to Group_Video_. In current generation VR, handling is an abstraction of reality that simplifies the physical behaviour and mechanical interaction, and haptic feedback is absent.

Unlike those who viewed a video of the exact real-world behaviour of the catheter and sterile gloves, those exposed to the VR environment were unable to gain an effective understanding of the real-world behaviour of these tools from the virtual abstraction of them. The virtual fidelity of the gloves and the catheter could be improved, and haptic feedback could be included to give a more realistic representation of fine motor skills. However, at the current stage of VR technology there is no standardised way to integrate haptic feedback [42] as seen in other domains such as dentistry or surgery where custom-built specialised hard- and software are developed for these tasks [35, 43]. Simulating highly realistic physical behaviour and realistic interaction with tools such as catheters or gloves in VR is complicated and resource-intensive. In contrast, creating an effective video training is easier and less resource-intensive. They have been shown to be effective in psychomotor skills training [28].

#### Simulation focus

The real-world video clips that were present in the VRhigh version did not lead to better skill performance. They were often not noticed, or there was not enough time to concentrate on them according to focus group feedback, perhaps they led to a cognitive overload. A lesson learned is to limit the information presented in VR and focus on the most important aspects for specific learning objectives. Different focuses could be set through different modes with different learning objectives.

#### Interaction fidelity

Group_VRhigh_ was not as satisfied with their simulation as Group_VRlow_, likely because the hand-tracking feature was perceived as not intuitive and even frustrating by the users; tracking was occasionally lost and gestures for grabbing objects had to be performed in a very specific manner that felt unrealistic. In current VR technology, hands are more often used like controllers, e.g. performing pinching movements or pointing, rather than realistic hand motions. Hand tracking did not lead to a higher fidelity of interaction, but rather to unmet expectations and frustration. However, there were some individuals who had never used a controller or something similar before, who stated that they preferred the hand-tracking system. Since this is a rare case, we recommend a controller-based system – at least until further progress in hand-tracking technology has been made.

In a previous pilot study, we evaluated the technology acceptance of an earlier VR training simulation for ETS [26]. Compared to the newer simulation of this study, the old one had lower interaction fidelity and fewer overall interaction requirements. Despite these limitations, participants were very satisfied with the simulation. This suggests that simplicity may be positively associated with user satisfaction. On the other hand, knowledge and skill acquisition appear to benefit from more interactive and high-fidelity interaction VR simulations [44]. In order to satisfy ease of use and perceived usefulness as well as effectiveness in improving knowledge and skill, VR interventions must be carefully balanced in terms of interaction complexity and interaction fidelity. Too little complexity, for example automatically triggering all interactions in VR by just touching in a point-and-click manner, could lead to bad learning results, as less critical thinking is needed and actions may not be fully comprehended. Whereas a high interaction complexity using multiple buttons and the controller joysticks to simulate different types of interactions could lead to better learning results but can also frustrate users if it is too complex.

### Generalisability limitations and future research

Although some meta-analyses combine various VR simulations into a single pool, the differences among them — hardware (e.g., HMDs, 2D screens, etc.), input systems (e.g., controllers, hands, haptic devices, etc.), the degree of interactivity, guidance, and feedback in the learning simulation — are vastly different. These differences are especially notable in the rapidly evolving field of VR technology and learning simulations.

Consequently, making general statements about VR’s efficacy is difficult. An assertion such as “VR was worse than a video tutorial for learning the skill of ETS; it should, therefore, not be used” is inaccurate and does not show the full picture. A more appropriate statement would be: “Our particular VR simulation was not as effective as the video tutorial for learning the ETS skill. What could be the reason for this, and how can we improve the simulation?” Based on this, the following research queries emerge: (1) how effective is VR generally in achieving various learning objectives; and (2) how to design VR simulations to successfully reach their intended goals and (3) how to effectively implement them into education.

From the authors’ perspective, future research should focus more on the second query, in order to answer questions about effective didactic design and form universal guidelines and principles. The first and third query can be answered better when we utilise the potential of the technology more effectively.

### Environmental limitations

Circumstances of COVID-19 complicated access to nursing schools, which is why we had to include two non-nursing schools, i.e. trainees for anaesthetic technologist.

Due to time constraints, the participants were only able to run through the VR scenario once. Another one or two repetitions might have improved their OSCE performances, since many participants used VR for the first time.

The number of participants in the quantitative and qualitative results do not match, since there was a group that received multiple interventions which we decided to exclude from the quantitative analysis, since a factorial experiment would not have been adjusted to our study design and power calculation and would have required a larger sample size as well as different statistical analysis.

As all participants received the same short theory presentation prior to interventions, this could have influenced the outcomes of the knowledge tests to be in a similar score range, not highlighting the differences between the interventions enough.

Moreover, Group_Video_ eventually received a VR intervention prior to the T2-knowledge-test and the response rate was low, which makes comparability of knowledge retention questionable.

Finally, although the observers for the OSCE were trained individuals, their ratings could have been subjectively influenced.

## Conclusions

Three different learning interventions were compared regarding the educational outcomes, acquisition and retention of knowledge, skill performance, cognitive learner satisfaction, and technology acceptance.

All groups were successful in acquiring knowledge and there were no significant differences between them. This result shows that VR could be used for students to acquire knowledge in the nursing domain to a similar capacity as video training. Since the return rate for the knowledge retention test was too low, no evidential statement can be made.

The video intervention was more successful than both VR interventions in conveying the practical skills of ETS. Possibly, the VR simulation users were too preoccupied getting to know an unfamiliar technology rather than focusing on the procedure. Furthermore, the abstract and simplified behaviour in terms of physics, instrument behaviour and use, and the absence of haptic feedback, made it more difficult to transfer the VR experience into the real-world setting. In contrast, a video allows the student to observe the procedure being carried out by an expert including all the details and fine motor skills. The production of a video requires fewer resources and is cheaper.

Therefore, we recommend that researchers should examine the use case thoroughly and consider alternatives prior to developing a VR simulation training. Further research on comparing the effectiveness of video and VR training in nursing disciplines is also necessary.

Perhaps VR is not ideal for practising practical and psychomotor skills at the moment, but it has the potential to increase learner satisfaction, motivation and confidence, as well as to prepare for practical training in a skills lab. With more knowledge on how to design effective educational VR simulations and improved haptics, this could change in the future. For now, we recommend a blended approach of video training to learn basic theory knowledge, followed by VR training for acquiring procedural knowledge, as well as training on manikins to perfect psychomotor skills.

## Data Availability

The material and data that support the findings of this study are available from the corresponding author upon request.

## List of abbreviations

VR: Virtual Reality
ETS: Endotracheal suctioning
OSCE: Objective structured clinical examination
ANOVA: Analysis of variance
HSD: Honest significant difference
ABC-SAT: Affective- behavioural-cognitive-satisfaction
VR-HAM: Virtual reality hardware acceptance model
M: Mean
Mdn: Median
SD: Standard deviation
